# Circadian and ultradian oscillations in bilateral rhythms of the crayfish chelipeds

**DOI:** 10.3389/fnint.2022.876137

**Published:** 2022-10-20

**Authors:** José A. Viccon-Pale

**Affiliations:** Laboratorio de Fisiología y Comportamiento Animal, Departamento El Hombre y su Ambiente, Universidad Autónoma Metropolitana Unidad Xochimilco, Ciudad de México, Mexico

**Keywords:** *Procambarus clarkii*, Birnbaum–Saunders distribution, gamma distribution, Inverse Gaussian distribution, Laplace distribution, smallest extreme value distribution, Weibull distribution

## Abstract

Circadian systems are composed of multiple oscillatory elements that contain both circadian and ultradian oscillations. The relationships between these components maintain a stable temporal function in organisms. They provide a suitable phase to recurrent environmental changes and ensure a suitable temporal sequence of their own functions. Therefore, it is necessary to identify these interactions. Because a circadian rhythm of activity can be recorded in each crayfish cheliped, this paired organ system was used to address the possibility that two quasi-autonomous oscillators exhibiting both circadian and ultradian oscillations underlie these rhythms. The presence of both oscillations was found, both under entrainment and under freerunning. The following features of interactions between these circadian and ultradian oscillations were also observed: (a) circadian modal periods could be a feature of circadian oscillations under entrainment and freerunning; (b) the average period of the rhythm is a function of the proportions between the circadian and ultradian oscillations; (c) the release of both populations of oscillations of Zeitgeber effect results in the maintenance or an increase in their number and frequency under freerunning conditions. These circadian rhythms of activity can be described as mixed probability distributions containing circadian oscillations, individual ultradian oscillations, and ultradian oscillations of Gaussian components. Relationships among these elements can be structured in one of the following six probability distributions: Inverse Gaussian, gamma, Birnbaum–Saunders, Weibull, smallest extreme value, or Laplace. It should be noted that at one end of this order, the inverse Gaussian distribution most often fits the freerunning rhythm segments and at the other end, the Laplace distribution fits only the segments under entrainment. The possible relationships between the circadian and ultradian oscillations of crayfish motor activity rhythms and between the probability distributions of their periodograms are discussed. Also listed are some oscillators that could interact with cheliped rhythms.

## Introduction

Since the first records of the locomotor activity of crayfish, variations in rhythmic patterns were observed, which opened the possibility of the presence of two or more oscillations in them. Under lighting and temperature conditions somewhat like those of their place of origin, these organisms presented three types of curves on the kymograph paper, one during the day and two at night (Szymanski, 1918). In the European crayfish, under prolonged darkness, there was a nocturnal activity distributed in irregular periods (Kalmus, 1938). Also under constant darkness, about half of the animals (*Orconectes virilis*) showed a unimodal rhythm, with peaks of activity at noon or midnight, and the other half a bimodal rhythm, with peaks at dawn and dusk; although the type of rhythm was generally constant for any individual, the time and duration of the activity could vary from day to day and one type of rhythm could change to another (Schallek, 1942). In other experiments, with the same crayfish (*O. virilis*) held in continuous darkness, the activity occurs mainly at night for the first few days, then becomes irregular, so that half of all peaks occur during the day (Roberts, 1944). With other crayfish (*Faxonella clypeata*), two different types of records were obtained, one type was produced by more active specimens between midnight and noon and the second type was produced between noon and midnight (Fingerman and Lago, 1957).

At the Cold Spring Harbor Symposium on Quantitative Biology, Colin S. Pittendrigh (1960) pointed out that we are faced with the conclusion that the organism comprises a population of almost autonomous oscillatory systems. He came to this conclusion from the model of the 2-oscillators for rhythms, suggested when Brown and Webb (1949) studied the daily rhythms of the fiddler crab, *Uca*, Pittendrigh et al. (1958) the eclosion rhythm in the fruit fly, *Drosophila* and Harker (1960) the activity rhythm in the cockroach, *Periplaneta*. Although the rhythms of the crayfish are not as direct antecedents of this approach by Pittendrigh (1960), a year later, Brown (1961) published the results of his reexamination of previously published data (Park et al., 1941) of the activity of a crayfish (*Orconectes pellucidus*) collected in Mammoth Cave, KY, United States. The activity of this cave crayfish was present at a rate of 24 h, with a minimum, approximately, at 9 a.m. and a maximum around 7 p.m, activity from 8 to 10 a.m. was less than half that from 6 to 8 p.m. (Brown, 1961). In other experiments with the same cave crayfish, under conditions of constant darkness and temperature, three animals were apparently regular in their rhythmicity and three were irregular or apparently random, of the first three, two showed statistically significant circadian periodicity (Jegla and Poulson, 1968). The crayfish (*Procambarus clarkii*) installed under a light cycle (LD 12:12), characteristically showed a rhythm, one of the activity peaks, the “lights on” peak, lasted approximately 10–30 min and was synchronized with the onset of light, the second peak of activity, the “light off” peak, occurred approximately 10–30 min after the onset of the dark phase of the photoperiod (Page and Larimer, 1972). In constant darkness (DD), this crayfish maintained a unimodal, free-oscillating circadian rhythm, involving only the peak of “lights out” activity (Page and Larimer, 1972). In the locomotor activity of the crayfish (*P. clarkii*), under LD, two peaks were observed during the night phase of the cycle, while the rest of the activity was characteristic of the diurnal phase, under DD or LL, a freerunning rhythm with a periodicity of 22.3 ± 0.84 h in DD and 24.8 ± 0.27 h in LL (Fernández de Miguel and Aréchiga, 1994). In the cave crayfish (*Procambarus cavernicola*), the freerunning period under DD was 23.2 ± 0.7, under LL (50 lux) was 20.1 ± 0.7 h and under the entrained regimen (12:12, LD), the period of the rhythm was 21.5 ± 0.6 h (de la O-Martínez et al., 2004).

Page and Larimer (1975) reported the occurrence of two ultradian oscillations in the circadian rhythms exhibited in the motor activity of the first and fourth legs of the crayfish *P. clarkii*. Under light-dark cycles, this activity showed “lights-on” and “lights-off” peaks, and under constant darkness, a unimodal circadian rhythm was maintained, involving only the “lights-off” peak. Another crayfish, *Procambarus bouvieri*, also exhibited a bimodal circadian rhythm of leg activity under constant light or darkness (Fuentes-Pardo and Inclan-Rubio, 1981; Fuentes-Pardo and Inclán-Rubio, 1987). From the phase shifts in the circadian rhythms of the ipsilateral legs of the crayfish *Procambarus digueti* caused by a single light pulse, three curves were constructed, these suggested that the chelipeds have a different circadian rhythm than the legs (Viccon-Pale and Fuentes-Pardo, 1994). In a subsequent investigation (Viccon-Pale et al., 1997), the effects of different lighting conditions on the simultaneously recorded activity rhythms of the two chelipeds were compared. The results raised the possibility that a system of quasi-autonomous oscillators showing circadian and ultradian oscillations underlies the activity rhythm in each of the cheliped. The goal of the present study was to address this possibility.

Studies on the interactions between ultradian and circadian oscillations outline three possible relationships as follows: (a) The circadian rhythm is established by ultradian oscillator coupling; (b) ultradian and circadian oscillators exist independently in organisms; (c) ultradian rhythms originate from the desynchronization of a population of circadian oscillators (Lazopulo and Syed, 2016). To assess the extent of these interactions, when periodogram analysis was used to determine the periodic components in the rhythm segments, the periods that were examined ranged from 0.01 to 30.0 h, the periods from 0.01 to 17.99 h were assigned to the ultradian range, and the periods from 18.00 to 30.00 h were assigned to the circadian range. Then, the models that best fit the probability distributions of the periodograms were considered, as well as the links of these distributions with the entrainment and freerunning patterns of the rhythms.

## Materials and methods

### General experimental conditions

The 21 male crayfish, *P. clarkii*, from the experiments, were purchased from a local animal dealer. In the laboratory, they were kept in individual aquariums under ordinary lighting and temperature conditions. Their diet, before being placed in the experiments, consisted of pieces of boiled carrots and small portions of meat. In each experiment the animals were kept under a dark-light cycle (DL, 12:12 h) for 6 days for entrainment (En) and then exposed to constant white light (LL) for another 6 days to allow freerunning (Fr) rhythms. All experiments began with a dark phase, starting at 18:00 h. The experimental light intensity was 9 lum/sq, and the temperature was 17°C.

### Recording spontaneous activity

No statistical methods were used to determine the sample size. The results were obtained from 21 simultaneous records of the spontaneous activity of the left cheliped (Lc) and the right cheliped (Rc) of the crayfish. For the simultaneous recording of the spontaneous activity of the two chelipeds (Viccon-Pale et al., 1997), the crayfish were attached to a clamp using a harness made of acrylic, a piece of cork and thread, and each cheliped was connected separately, by means of a thread, to a different force transducer (Narco-Biosystems, F-2000). The activity signals converted into electronic signals by the transducer were sent to a transducer coupler (Narco-Biosystems, Type 7173), each connected to an amplifier (Narco-Biosystems, Type 7070). Analogical signals (range/mode 10 V) from the amplifiers were digitalized through PC-based data acquisition (Measurement Computing, USB-1208FS). The acquisition rate of the activity signals was 1 data point per minute. To eliminate bias caused by the operation of the information channels, the channels were exchanged between chelipeds from one experiment to the next. Activity signals were integrated over 30 min periods.

### Data processing

Each time series data of the activity was smoothed by calculating the 5-term moving average (Schuster, 1898). All statistical analyses were performed using the Statgraphics software (Centurion XVI, 16.0.07). The time series were divided into two segments, each containing 288 data points, with one segment corresponding to 6 days of the entrainment regimen and the other corresponding to 6 days of freerunning conditions. To compare the temporal features, the activity of each segment was normalized to 100 arbitrary units (a.u.). Periodogram analysis was used to determine the periodic components in time series segments (Schuster, 1898; Enright, 1965). Because the width and amplitude of the primary peak and the periods corresponding to the additional peaks in the periodogram depend on the amount of data analyzed (Enright, 1965), the number of input data points from the segments remained constant at 288. The step size was 0.50 h. As it was intended to evaluate the relationships between circadian oscillations and ultradian oscillations, the periods from 0.01 to 30.00 h were examined. Periods from 0.01 to 17.99 h were assigned to the ultradian range, and those from 18.00 to 30.00 h were assigned to the circadian range. The ordinate scales of the periodogram table varied for each segment; therefore, we normalized them to 100 units. Then, periodograms were reconstructed in a column of an electronic spreadsheet, repeating the periods as many times as indicated by their corresponding ordinates. These period frequency distributions provided information on the average periods, standard deviations (SD), and modal periods of the rhythms.

### Probability distributions

To study the relationships between circadian and ultradian oscillations, probability distribution models were fitted to periodograms. Previously, through the Shapiro–Wilk test, it was confirmed that the distribution showed characteristics of a normal distribution (95% confidence). Then, according to the log-likelihood statistics, the model that best fit the distribution was selected. However, because the only parameters of the uniform model were the lower limit and upper limit, the model with the second-best fit was chosen. The Kolmogorov–Smirnov test was performed to determine if the selected model adequately fit the corresponding probability distribution. Fitting the probability distributions yielded the estimated parameters of the model period, model mode, shape, and scale.

### Occurrence of probability distributions under freerunning conditions

Because the distributions can reflect different operation of the oscillators under entrainment or oscillation, it was considered that it could be fruitful to calculate the probability that one of the distributions occurs in a rhythm segment under conditions of freerunning. For this, the equation *P*(*Fr*) = *NFr*/(*NFr* + *NEn*), where *NFr* is the number of times the distribution occurred in segments under Fr and *NEn* is the number of times it occurred in segments under En. In Table 4, the probability distributions are grouped and ordered according to the mean of the average periods. To obtain information about the associations of these means and the model period means, as well as the modal period means and the model mode means, batches of these groups of means were compared using the multiple range tests with 95% significance.

### Gaussian components

The probability distributions of the periodograms were divide into parts or simple frequency curves (Pearson, 1895) through the method of Bhattacharya (1967). Using FiSAT software (Gayanilo et al., 2005), each Gaussian component was identified as an average period class with a class interval of 0.5 h. In addition, averages, SD, coefficients of determination (R2) for each average period class and the value of the separation index (SI) for each pair of adjacent classes were calculated.

## Results

### Examples of simultaneous recordings

**FIGURE 1:**
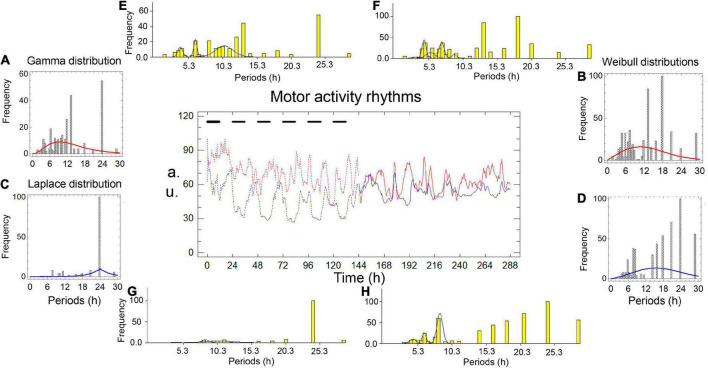
Motor activity rhythms of the chelipeds of a crayfish under entrainment and during freerunning. In the central graph, the independent variable is time, measured in hours; the dependent variables are the amount of spontaneous activity, measured in arbitrary units (a.u.). The black bars represent the dark phase of the dark-light cycle. The record from the left cheliped (Lc) is the red line, and the record from the right cheliped (Rc) is the blue line. The broken lines represent the entrained rhythms (En) under dark-light cycles (DL, 12:12) for 6 days, and the solid lines represent the freerunning rhythms (Fr) under constant light (LL) for another 6 days. Panels **A–D** are periodograms and models of the probability distributions. Panels **E–H** are periodograms and Gaussian components.

**FIGURE 2:**
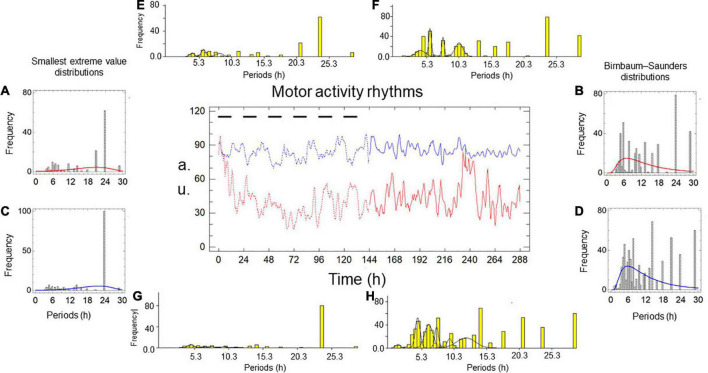
Motor activity rhythms of the chelipeds of a crayfish under entrainment and during freerunning. In the central graph, the independent variable is time, measured in hours; the dependent variable is the amount of spontaneous activity, measured in arbitrary units (a.u.). The black bars represent the dark phase of the dark-light cycle. The record from the left cheliped (Lc) is the red line, and the record from the right cheliped (Rc) is the blue line. The broken lines represent the entrained rhythms (En) under dark-light cycles (DL, 12:12) for 6 days, and the solid lines represent the freerunning rhythms (Fr) under constant light (LL) for another 6 days. Panels **A–D** are periodograms and models of the probability distributions. Panels **E–H** are periodograms and Gaussian components.

Figures 1, 2 and Tables 1, 2 show the results of two simultaneous recordings of the rhythm of motor activity of crayfish chelipeds under entrainment and freerunning conditions.

**TABLE 1:** Models and parameters of the records presented in Figure 1.

A				B			
	
Oscillation states	Entrainment	Comparison	Entrainment	Oscillation states	Entrainment	Comparison	Freerunning
Chelipeds	Left		Right	Chelipeds	Left		Left
Distribution Models	Gamma		Laplace	Distribution Models	Gamma		Weibull
Counts	275	>	152	Counts	275	<	543
Average periods (h)	13.05	<	20.66	Average periods (h)	13.05	=	13.07
Modal periods (h)	24.00	=	24.00	Modal periods (h)	24.00	>	18.00
SDs	6.87	=	6.25	SDs	6.87	=	6.77
Shapes	3.41			Shapes	3.41		2.05
Scales	0.26		0.27	Scales	0.26		14.18
Model Means (h)			24.00				

**C**				**D**			
	
**Oscillation states**	**Entrainment**	**Comparison**	**Freerunning**	**Oscillation states**	**Freerunning**	**Comparison**	**Freerunning**

Chelipeds	Right		Right	Chelipeds	Left		Right
Distribution Models	Laplace		Weibull	Distribution Models	Weibull		Weibull
Counts	152	<	514	Counts	543	>	514
Average periods (h)	20.66	>	16.96	Average periods (h)	13.07	<	16.96
Modal periods (h)	24.00	=	24.00	Modal periods (h)	18.00	<	24.00
SDs	6.25	<	7.62	SDs	6.77	<	7.62
Shapes			2.43	Shapes	2.05		2.43
Scales	0.27		19.16	Scales	14.80		19.16
Model means (h)	24.00						

**TABLE 2:** Models and parameters of the records presented in Figure 2.

A				B			
	
Oscillation states	Entrainment	Comparison	Entrainment	Oscillation states	Entrainment	Comparison	Freerunning
Chelipeds	Left		Right	Chelipeds	Left		Left
Distribution models	Smallest Extreme Value		Smallest Extreme Value	Distribution Models	Smallest Extreme Value		Birnbaum-Saunders
Counts	155	<	166	Counts	155	<	438
Average periods (h)	17.07	=	18.37	Average periods (h)	17.07	>	13.84
Modal periods (h)	24.00	=	24.00	Modal periods (h)	24.00	=	24.00
SDs	8.03	=	7.90	SDs	8.03	=	8.32
Scales	6.24		5.39	Shapes			0.67
Mode models (h)	20.86		21.92	Scales	6.24		11.28

**C**				**D**			
	
**Oscillation states**	**Entrainment**	**Comparison**	**Freerunning**	**Oscillation states**	**Freerunning**	**Comparison**	**Freerunning**

Chelipeds	Right		Right	Chelipeds	Left		Right
Distribution Models	Smallest Extreme Value		Birnbaum-Saunders	Distribution Models	Birnbaum-Saunders		Birnbaum-Saunders
Counts	166	<	643	Counts	438	<	643
Average periods (h)	18.37	>	12.52	Average periods (h)	13.84	>	12.52
Modal periods (h)	24.00	>	14.4	Modal periods (h)	24.00	>	14.40
SDs	7.90	=	7.91	SDs	8.32	=	7.91
Shapes			0.71	Shapes	0.67		0.71
Scales	5.39		9.97	Scales	11.28		9.97
Mode models (h)	21.92						

Given the variability in recording characteristics from a single experiment and between recording properties from different experiments, the results given in these figures do not capture the full variability in the enriched data sets. Despite these differences, the size of the samples, their grouping and analysis made it possible to detect regularities described in the present study.

Under entrainment in Figure 1. The activity rhythms responded to the zeitgeber with three types of bilaterally asymmetric reactions: (a) the shape of the rhythm of the left cheliped (L-rhythm) showed a roughly sinusoidal waveform, while the rhythm of the right cheliped (R-rhythm) showed an imperfect square waveform; (b) the gamma distribution model fit the periodogram of the L-rhythm (Figure 1A), while the Laplace distribution model fit the periodogram of the R-rhythm (Figure 1C); (c) there was a significant difference between the average periods of the two rhythms (Table 1A). A bilaterally symmetrical response to the zeitgeber was observed, the modal periods of the rhythms of both chelipeds had the same value at 24.00 h (Table 1A).

From entrainment to freerunning (Figure 1). In the L-rhythm, the roughly sinusoidal waveform remained, and the gamma distribution became a Weibull distribution (Figures 1A,B), the total frequency of the periods increased from 275 to 543 units, there were no significant changes in the average periods and the value of the modal periods decreased from 24.00 to 18.00 h (Table 1B). Although the number of oscillations remained at 22 units, the number of Gaussian components increased from three to five, the number of individual ultradian oscillations increased from three to six, and the number of circadian oscillations remained at four (Figures 1E,F). In the R-rhythm, the imperfect square wave became more imperfect, and the Laplace distribution was transformed into a Weibull distribution (Figures 1C,D), the total frequency of the periods increased from 152 to 514 units, the average period decreased from 20.66 to 16.96 h, and the value of the modal period remained at 24.00 h (Table 1C). The number of oscillations ranged from 13 to 20 units, the number of Gaussian components increased from two to three, the number of individual ultradian oscillations ranged from zero to four, and the number of circadian oscillations remained at four (Figures 1G,H).

For freerunning rhythms (Fr) (Figure 1), as a result of the rhythmic transformations in the transition from entrainment to freerunning, three asymmetries and two symmetries were observed: (a) The L-rhythm continued to show a roughly sinusoidal waveform, and the R-rhythm showed a more imperfect square waveform (Figure 1); (b) the average period of the L-rhythm was shorter than that of the R-rhythm; (c) the modal period of the L-rhythm was shorter than that of the R-rhythm; (d) the two rhythms had almost the same number of periods; (e) the model that fit the periodograms of both rhythms showed a Weibull distribution (Table 1D and Figures 1G,H).

Under entrainment in Figure 2. The activity rhythms responded to the zeitgeber with four types of bilaterally symmetric reactions: (a) the L-rhythm and R-rhythm shapes are imperfect square waveforms (Figure 2); (b) the distribution of the smallest extreme value fits the periodograms of both rhythms (Figures 2A,C); (c) there was no significant difference between the average periods; and (d) the modal periods of the rhythms have the same value (Table 2A).

From entrainment to freerunning (Figure 2). In both rhythms, the imperfect square wave became a roughly sinusoidal waveform, and the smallest extreme value distribution became a Birnbaum–Saunders distribution (Figures 2A–D). In the L-rhythm (Figures 2A,B,E,F and Table 2B), the total frequency of the oscillations increases from 155 to 438 units, the average period decrease from 17.02 to 13.84 h, the value of the modal period remained at 24 h, the number of oscillations per period increased from 20 (four grouped between three Gaussian components, four independent ultradian, and four circadian) to 26 (19 grouped between four Gaussian components, three independent ultradian, and four circadian). In the R-rhythm (Figures 2C,D,G,H and Table 2C), the total frequency of the oscillations increases from 166 to 643 units, the average period decreases from 18.37 to 12.52 h, the value of the modal period decreases from 24 to 14.40, the number of oscillations per period increases from 20 (four grouped between three Gaussian components, four independent ultradian and four circadian) to 28 (24 grouped between six Gaussian components and four circadian).

For the freerunning rhythms (Figure 2), as a result of the rhythmic transformations in the transition from entrainment to freerunning, two types of symmetries and two types of asymmetries were observed: (a) the shape of the L-rhythm and the R-rhythm showed approximately sinusoidal waveforms, the Birnbaum–Saunders distribution fits the periodograms of both rhythms (Figures 2B,D), the average period of the L-rhythm is greater than that of the R-rhythm and the modal period of the L-rhythm is also greater than that of R-rhythm.

### Means of the average periods and means of the modal periods

Table 3 shows the means of the average periods and the means of the modal periods of the segments under entrainment and under freerunning of the L-rhythm and the R-rhythm. In this, the means of the average periods are ultradian and those of the modal periods are circadian. Bilateral symmetries of both means of the segments under entrainment and under freerunning appear. Ipsilateral asymmetries also occur in the means of the average periods under entrainment and freerunning; the means of the segments under entrainment are greater than under freerunning. In the means of the modal periods only an ipsilateral asymmetry occurs, it is in the segments of the R-rhythm; also, the mean of the segments under entrainment is greater than under freerunning. There is no statistical difference between the means of the modal periods of the L-rhythm.

**TABLE 3:** Results of the comparisons of the means of the four batches, with 21 data each, of the average periods and the modal periods (first row), under entrainment and freerunning (second row) of the two cheliped (fourth row for the rhythms of the left cheliped and sixth row for the rhythms of the right leg).

A: Means of the average periods (h)			B: Means of the modal periods (h)		
	
Entrainment		Freerunning	Entrainment		Freerunning
**Left chelipeds rhythms**					
18.05 ± 2.76	4.60 ± 1.66	13.45 ± 2.65	23.10 ± 2.73	=	20.27 ± 7.23
=		=	=		=
18.14 ± 2.78	3.84 ± 1.66	14.30 ± 2.64	24.29 ± 1.67	3.98 ± 3.07	20.31 ± 6.43
**Right chelipeds rhythms**					
Levene’s test = 0.08, *P*(0.97)>0.05			Levene’s test = 14.25, *P*(0.00)<0.05		
Least significant difference (LSD) at 95%					

The numbers in the two unlabelled columns represent the differences in means between the batches under entrainment and under freerunning.

**TABLE 4:** Probability distributions and average periods. The number of distributions (No.). *P*(Fr). Sources of data, periodograms, and models. Means of the average periods (AvPe), means of the modal periods (MoPe), shapes, and scales. Bs, number of bilateral symmetries under En | Fr.

Models	*P*(*Fr*)	Data source	MeAvPe. (h)	MeMoPe (h)	Shapes	Scales
Inverse Gaussian (7) Bs: 0| 0	0.86	Periodograms	11.45 ± 0.67	11.43 ± 1.21		
		Models	11.45 ± 0.67			3.15 ± 1.20
Gamma (10) Bs: 0| 0	0.90	Periodograms	12.60 ± 1.70	20.65 ± 7.77		
		Models			3.29 ± 0.74	0.27 ± 0.07
Birnbaum-Saunders (15) Bs: 0| 2	0.47	Periodograms	13.30 ± 1.26	22.49 ± 5.54		
		Models			0.74 ± 0.23	10.24 ± 2.11
Weibull (22) Bs: 0| 1	0.55	Periodograms	16.32 ± 2.52	23.34 ± 3.55		
		Models			2.62 ± 0.76	18.52 ± 2.58
Smallest Extreme Value (21) Bs: 5| 0	0.14	Periodograms	18.51 ± 1.46	24.00		
		Models		21.86 ± 1.78		5.56 ± 0.63
Laplace (9) Bs: 0| 0	0	Periodograms	21.04 ± 1.04	24.00		
		Models	24.00			0.39 ± 0.26

### Probability distributions

Six probability distributions were fitted to cheliped rhythm periodograms: Inverse Gaussian, Laplace, gamma, Birnbaum–Saunders, smallest extreme value, and Weibull. These are mixed probability distributions composed of circadian oscillations, individual ultradian oscillations, and ultradian oscillations of Gaussian components (Figures 1, 2). In general, the circadian oscillations include the primary peak of the periodograms, which should correspond to the modal period, while the rest of the circadian and ultradian oscillations should correspond to the additional peaks of the periodograms.

The six probability distributions of the cheliped rhythms were distributed among the 84 activity rhythm segments of the L- or R-cheliped, under entrainment or during freerunning, with the following proportions: Inverse Gaussian at 8.33%, Laplace at 10.71%, Gamma at 11.90%, Birnbaum–Saunders at 17.86%, smallest extreme value at 25.00%, and Weibull at 26.19%. Table 4 shows the probability distributions according to the mean of average periods.

## Discussion

Following Goldbeter (2017), biological rhythms can be interpreted as temporary dissipative structures. This concept introduced by Prigogine (1978) allows us to observe the changes in the structures of circadian and ultradian oscillations, which are described using probability distributions, such as fluctuations in the circadian rhythms of crayfish motor activity, which are entrained and maintained under freerunning conditions.

### Circadian and ultradian oscillations in cheliped rhythms

The presence of ultradian and circadian oscillations in the activity rhythms of the two crayfish chelipeds, both under entrainment and under freerunning reinforces the second hypothesis presented by Lazopulo and Syed (2016): ultradian and circadian oscillators exist independently in the organism. The following aspects of the interactions between circadian and ultradian oscillations were observed: (a) Circadian modal periods are a characteristic of circadian oscillations under entrainment and during freerunning; (b) the average period of the rhythm was a function of the proportions between circadian and ultradian oscillations; (c) the release of both populations of oscillations due to the zeitgeber effect resulted in the maintenance of or an increase in their number and an increase in their frequency during freerunning. This supports the hypothesis of relative independence and quasi-autonomy of the mechanisms involved in each oscillation (Winfree, 1975). Because the photoreceptors in the crayfish brain are sufficient to drive the rhythms of locomotor response to photic stimuli (Sullivan et al., 2009), perhaps these oscillators could be entrained in parallel, directly, and in different phase relationships using the external light-dark cycle (Winfree, 1975). These results align with those of previous studies. In periodograms of the locomotor rhythm of the crayfish *P. clarkii*, the effect of monochromatic light pulses, retinal ablation, and changes in illumination and temperature on circadian oscillations have been associated with the primary peak (Miranda-Anaya and Fanjul-Moles, 1997; 13). However, the effect of these stimuli on the additional peaks (Miranda-Anaya and Fanjul-Moles, 1997; Palma-Anzures et al., 2012) can also be observed, which can be linked to ultradian oscillations.

### Anatomical and physiological substrates of formal asymmetries

With the intention of starting the identification of possible anatomical and physiological substrates of the formal asymmetries identified in the bilateral recordings of the rhythms of the legs of crayfish, some asymmetries observed in the two caudal photoreceptors, which participate in the entrainment of the rhythms of activity (Fuentes-Pardo and Inclán-Rubio, 1987), can be considered. Such asymmetries are shown in (a) light- and dark-induced spontaneous firing rates; (b) the differential effect of temperature both in the dark and during light-induced activity (Pacheco-Ortiz et al., 2018); (c) the firing rate in response to pulses of monochromatic, blue, and green light (Sánchez-Hernández et al., 2018).

### Probability distributions, entrainment, and freerunning

The first two lines of Table 4 show the inverse Gaussian and gamma distributions. Schrödinger (1915) provided the probability distribution of the first passage time (inverse Gaussian) in Brownian motion (Folks and Chhikara, 1978), which consists of random fluctuations. In this investigation, it was found that the rhythms most frequently described using the inverse Gaussian distribution are those that are under freerunning [*P*(*Fr*) = 0.86] and that there are no significant differences between the mean of average periods, the mean model periods (which are, in fact, the same), and the mean modal periods, which are ultradian and characterize these rhythms (Table 4). Based on these results and recently mentioned antecedents, the possibility arises that the rhythms under freerunning conditions described using the inverse Gaussian distribution are stochastic processes. Meanwhile, the rhythms described using the gamma distribution, also with higher occurrence under freerunning conditions [*P*(*Fr*) = 0.90], with a mean circadian modal period of 20.65 ± 7.77 h, are rhythms that are already structured as circadian oscillations. Of particular interest, in the domain of statistics, the inverse Gaussian distribution and the gamma distribution share asymptotic convergence to normality and their density function graphs are similar (Folks and Chhikara, 1978). In the present study, experimental results showed that the means of the average periods of inverse Gaussian distribution and the gamma distribution were ultradian, while the mean of the modal periods in the first distribution was ultradian and in the second circadian (Table 4).

The second and third rows of Table 4 are occupied by the Birnbaum–Saunders and Weibull distributions, respectively. In the two distributions, the means of the average periods remain at ultradian magnitudes, and the means of the modal periods were circadian. The *P*(*Fr*) of the Birnbaum–Saunders distribution was 0.47, and that of the Weibull distribution was 0.55. In other words, the first distribution was already more widespread in the entrained rhythms than in the Fr, and the presence of the latter was the same between the two rhythm categories. Of the six distributions that fit the cheliped rhythms, the Weibull distribution was the most widely observed among the different batches, providing a good fit in several cases. About eight decades ago, Weibull introduced a new distribution function to describe the strength properties of materials and then used it for the statistical representation of the fatigue failure in solids and several distribution problems in other fields (Weibull, 1949). Although the distribution was sometimes complex, following the same formula with different parameters, Weibull (1949) was able to analyze the distribution in its components. For example, some samples of the Radiolaria size presented a distribution composed of two simple distributions. In this study, probability distributions were divided into Gaussian components (Bhattacharya, 1967), individual ultradian oscillations, and circadian oscillations. On another scale, the Weibull distribution effectively described the intervals of spontaneous electrical activity of the neuromuscular junction recorded under seven different calcium concentrations (da Silva et al., 2020).

The smallest extreme value is presented in the fourth row of Table 4 and the Laplace distribution occupies the fifth row of the same table. The *P(Fr)* of the smallest extreme value distribution was 0.14, and that of the Laplace distribution was zero. In other words, after the Weibull distribution, the arrangement of probability distributions that describe circadian rhythms was more likely to show an arrangement for the description of them under entrainment. In harmony with this arrangement, in the responses of rhythms to the zeitgeber, there were five bilateral symmetries with the smallest extreme value distributions. In four of these symmetries, there were no statistically significant differences between the means of the average periods and those of the modal periods (Table 4). In the theory of extreme values, the mode, instead of the mean, is entered directly as one of the location parameters and therefore is preferred over the mean (Gumbel, 1954). Despite this, in Table 4 it was noted that the average means of the average periods of the symmetries was 18.47 ± 1.11 h, which presented a phase difference with the modes and a zeitgeber cycle of 6 h. Therefore, it was interpreted as this difference was due to the stability of the entrainment and interaction of the oscillators. In contrast, the Laplace distribution, occasionally called the double exponential distribution (Kotz et al., 2001), illustrated an unstable effect of the zeitgeber on circadian and ultradian oscillations. The mean of average periods was 21.04 ± 1.05 h, while the mean of the model periods was 24.00 h, and the mean of the modal periods was 24.00 h. Magnitudes of these parameters, which are location parameters, were the same as those of the zeitgeber cycle duration, which was 24.00 h; therefore, the phase difference between these three parameters and the mean of average periods was 2.47 h. These results, as well as the fact that the Laplace distribution occurred only in one or another rhythm of the chelipeds and prevailed only under the entrainment, could represent a state of maximum entrainment, although unstable, from the oscillator to the zeitgeber, which is only induced and maintained by the presence of the zeitgeber.

### The presence of at least two oscillator systems

The simultaneous recordings of the activity rhythms in the crayfish chelipeds and the description of these rhythms through their probability distributions opened the possibility of answering the crucial question of whether the circadian rhythmicity in the chelipeds entrainment is maintained by a single pacemaker or a master clock located in the brain, or by several relatively independent oscillators (Block and Page, 1978; Sullivan et al., 2009). Given that the entrainment regime and freerunning conditions were constant and the response of the activity rhythms of the two crayfish chelipeds could adopt one of six or five probability distributions, such adoptions may originate through the internal processes of at least two oscillator systems. The location of circadian oscillators in bilaterally symmetric structures, such as the chelipeds of crayfish (Viccon-Pale et al., 1997), increases the possibility that a quasi-autonomous oscillator may reside in them (Block and Page, 1978).

In a model proposed for circadian rhythms in *Neurospora*, the appearance of several modes of dynamic behavior, such as the quasi periodicity, synchronization, period duplication and chaos depend on the forced waveform; the domain of entrainment grows at the expense of the domain of chaos as the forcing function progressively goes from a square wave to a sine wave (Gonze and Goldbeter, 2000). In the activity rhythms of the crayfish chelipeds, in general, under freerunning, the activity rhythms show roughly sinusoidal waveforms and under entrainment may exhibits bilateral asymmetric responses of the waveforms to the Zeitgeber. In other words, under the same Zeitgeber, the rhythm of one cheliped can be displayed as a roughly sinusoidal waveform and the rhythm of the other cheliped is displayed as an imperfect square waveform; moreover, the more one form is defined, the less the other is defined. This raises the possibility that a self-contained oscillator may reside in each cheliped.

Table 3 shows some aspects of the responses of the means of the average periods, which could represent the ultradian oscillations and the means of the modal periods, which could represent the circadian oscillations, of the rhythms of both chelipeds, to the entrainment regime and to freerunning conditions, which support the hypotheses of relative independence and quasi-autonomy of the mechanisms involved in ultradian and circadian oscillations (Winfree, 1975). Under entrainment and freerunning, there are no statistically significant differences between the means of the average periods and their SD of the rhythms of the two chelipeds, on the contrary, the means under entrainment are greater than the means in freerunning (Table 3A); as if the latter were released from the Zeitgeber effects. Under the same entrainment regime and under the same freerunning conditions, there are no statistical differences between the means of the modal periods, but there are differences between the SD of the rhythms of the two chelipeds (Table 3B). While the mean in the R-cheliped is higher under entrainment than in the freerunning, in the L-cheliped there are no statistical differences between them (Table 3). This and the greater normal deviation of this than the right, suggest different responses of the L-chelipeds to the Zeitgeber and the possibility of different oscillatory systems in chelipeds. On the other hand, these results could be in line with those of other previous studies. In locomotor rhythm periodograms of the crayfish *P. clarkii*, associated with the effect of monochromatic light pulses, retinal ablation, and changes on illumination and temperature on the primary peaks (Miranda-Anaya and Fanjul-Moles, 1997; Palma-Anzures et al., 2012), effects on additional peaks can also be observed. If the primary peak of the periodograms were related to circadian oscillations and the additional peaks to ultradian oscillations, the effect of these zeitgebers and retinal ablation on ultradian oscillations could be observed.

In this work, most of the asymmetries registered under oscillation with the probability distributions support this hypothesis. When the crayfish were transferred from everyday laboratory conditions to the entrainment regime, the oscillatory systems of their chelipeds could choose one of the six probability distributions. One of them, the Laplace distribution, which could be associated with a stationary state of maximum entrainment, only occurred under this regimen and only in one of the two legs. Upon transition to the freerunning conditions, the options were reduced to five. One of them, the inverse Gaussian distribution, which has been applied to the description of stochastic processes (Folks and Chhikara, 1978), was recorded more frequently under freerunning conditions, and only occurred in one of the two legs. Because neither the last distribution (with ultradian mean of the average periods, modal periods, and model periods), nor the Laplace distribution (with a circadian mean of 21.04 h in the average periods and 24 h in the modal periods and model periods), showed symmetries in the simultaneous records under entrainment, the oscillations described by them could have promoted instabilities in the interactions of at least two oscillator systems. On the contrary, the distribution of the smallest extreme value with a circadian mean of 18.51 h in the average periods, 24 h in modal periods, and 21.86 h in model periods was displayed in five symmetries under entrainment, which may have revealed the stability of the oscillator interrelationships described using this distribution and its parameters.

### A possible system of oscillators

Schallek (1942) formulated a model in which “the activity of the limbs is inhibited by fibers passing down the optic nerve and crossing the mid-line of the brain. These conditions are filled by the fibers which form the optic chiasma (…). Impulses from these fibers seem to inhibit the activity of the crayfish during the quiet phase of its diurnal rhythm…that this activity can be modified by light, presumably through impulses arising in the retina (Schallek, 1942: 163).” Kropp and Enzmann (1933) Covering one eye and exposing the uncovered eye to light, they reported that the movements of the leg of crayfish “on the side of the exposed eye are more frequent and are of greater amplitude (Kropp and Enzmann, 1933: 907).” It was based on his experiments that Schallek (1942) formulated that darkening of the eye decreases the activity of inhibitory fibers that cross the midline of the body and that such unilateral activity would be difficult to explain on an endocrine basis. Schallek deduced that “if impulses from inhibitory centers control the quiet phase of the activity rhythm, these impulses must stop during the active phase (Schallek, 1942: 164).” Prosser (1934) had discovered spontaneous activity in the crayfish nervous system, so Schallek hypothesized that “there may be an intrinsic rhythm in the spontaneous activity of these inhibitory centers (Schallek, 1942: 164).” Now, in this work we present the observation that a light-dark cycle (12:12) inhibits the frequency of the ultradian oscillations and therefore increases the average period of the rhythm. It remains to be seen whether, according to Schallek’s (1942) model, the effect is contralateral. In addition, with the results obtained, since then to date, in different investigations, the possibility of improving this model has also been opened.

Until 1975, several lines of evidence had suggested “that the supraesophageal ganglion is the source of a driving oscillation(s) sufficient for generating circadian rhythms in decapods (Page and Larimer, 1975: 76).” An interpretation that Page and Larimer (1975: 76) made of their activity rhythm data was “that a circadian oscillation originates in the supraesophageal ganglion and is transmitted to the thoracic locomotor centers *via* axons in the circumesophageal connectives.” Results from a variety of experiments provided evidence that signals entrainment circadian rhythms of crayfish locomotor activity and electroretinogram (ERG) amplitude can be transmitted through an extraretinal pathway and that these extraretinal photoreceptors are in the supraesophageal ganglion (Page and Larimer, 1976). The results of Sullivan et al. (2009) suggest that the photoreceptor neuropils of the crayfish brain represent part of an entrainment pathway that synchronizes the rhythms of locomotor activity with photic stimuli, which may act in the absence of compound eyes and caudal photoreceptors, involving to the neuropeptide pigment-dispersing hormone (PDH) in these functions. However, since bilateral section of the optic tracts produced the same hyperactivity as eyestalk removal, Page and Larimer (1975) suggested that the release and/or production of the hormone that inhibits the activity is controlled by the brain, which leads to relationships between the eyestalk and the brain.

Sánchez and Fuentes-Pardo (1977) observed in isolated crayfish eyestalks under conditions of total darkness and constant temperature that the ERG voltage amplitude exhibited circadian variations and high-frequency oscillations that appeared to be related to circadian time. They proposed that these high-frequency cycles result from multiple oscillators, which when synchronized give rise to circadian rhythmic variation. Aréchiga and Rodríguez-Sosa (1998) recorded ERG of crayfish in isolated preparations of the retina and ganglionaris. “ERG amplitude varied in a circadian manner with a nocturnal acrophase and a period of 22–23 h in preparations kept in darkness (Aréchiga and Rodríguez-Sosa, 1998: 1819).”

Fuentes-Pardo and Inclan-Rubio (1981) obtained a correlation between the ERG and locomotor rhythms of crayfish. They observed a phase difference of 4 h in free walking. Changes in the frequency of the ERG-evoking flash alter both rhythms. With cerebral ganglion resection, circadian rhythmicity persisted in both rhythms, but there were some changes in their period. Simultaneous recordings 9 days after ganglion resection also showed loss of phase difference (Fuentes-Pardo and Inclan-Rubio, 1981). These results lead to the possibility that several feedback loops underlie the origin, maintenance, and coupling of these rhythms, both in the cerebroid ganglion and in the eyestalk. Agapito et al. (1995) report the presence of melatonin and the enzyme serotonin-N-acetyltransferase (SNAT) in the eyes (globe plus eyestalk) of the freshwater crayfish *P. clarkii*. Both melatonin and SNAT activity exhibit circadian variations, with their acrophase during the light phase and their nadir during darkness. The results of Valdes-Fuentes et al. (2011), using an isolated eyestalk–brain preparation, showed that photic stimulation of retina produced changes in both the amplitude and the frequency of spontaneous electrical activity in the protocerebral neuropils. In addition, electrical stimulation of the medial protocerebrum, particularly the protocerebral bridge, produced changes in the ERG that depended on both the time of day and the level of serotonin. This suggests that pathways between retina and protocerebral bridge seem to be serotonergic (Valdes-Fuentes et al., 2011).

Rodríguez-Sosa et al. (2008) documented two circadian rhythms for crayfish caudal photoreceptor electrical activity, spontaneous and light-induced discharge of action potentials. Under darkness, spontaneous activity varies in a circadian manner, with a period of 24.7 h. For light-induced activity, the firing rate varies rhythmically, the period is 24.24 h. An ultradian rhythm of a 12-h period was observed for both rhythms. The phase shift caused by temperature for these circadian rhythms depends on the time of application. In addition, the level of the serotonin receptor (5-HT1A) shows a diurnal rhythm in the 6th abdominal ganglion, with acrophase at twilight.

### Some of the pending issues

According to Aschoff (1960), the circadian period of nocturnal animals decreases when the intensity of constant lighting decreases. Since the crayfish, *P. clarkii*, is a nocturnal animal and was exposed to LL in this study, under DD, will the average period of the motor activity rhythms of its chelipeds decrease, compared to how they are here, under LL? In this work it was observed that the average period of the rhythm is a function of the ratios between the circadian and ultradian oscillations. Likewise, linked to this Aschoff rule and the results found, the questions that arise are, under DD, will the ultradian oscillations increase in number and/or frequency? And/or circadian oscillations will decrease in number and/or frequency? Another pending topic is that of the relationships between the response of the oscillations to changes in the duration of the zeitgeber and the photoperiod.

The preparation for the simultaneous recording of the circadian rhythms of the motor activity of the crayfish legs (Viccon-Pale et al., 1997) used in the experiments whose results have been presented in this work, may be used to test some hypotheses that may be derive from the relationships between the oscillators of the system outlined in the previous paragraphs and the record of other possible bilateral circadian rhythms and establish possible interactions between them. For example, that of the electromyographic (EMG) patterns of the walking leg muscles of *P. clarkii* itself (Tomina et al., 2013). Derivation of experimentally collected probability distribution interrelationships through probability theory is necessary.

## Data availability statement

The raw data supporting the conclusions of this article will be made available by the authors, without undue reservation.

## Ethics statement

This animal study was reviewed and approved by the Consejo de la División de Ciencias Biológicas y de la Salud, UAMX, Mexico, in its session 10/17 of October 10, 2017. Its update is in process.

## Author contributions

The author confirms being the sole contributor of this work and has approved it for publication.
